# Segmental Bayesian estimation of gap-junctional and inhibitory conductance of inferior olive neurons from spike trains with complicated dynamics

**DOI:** 10.3389/fncom.2015.00056

**Published:** 2015-05-21

**Authors:** Huu Hoang, Okito Yamashita, Isao T. Tokuda, Masa-aki Sato, Mitsuo Kawato, Keisuke Toyama

**Affiliations:** ^1^Department of Mechanical Engineering, Ritsumeikan UniversityShiga, Japan; ^2^ATR Neural Information Analysis LaboratoriesKyoto, Japan; ^3^Brain Functional Imaging Technologies Group, CiNetOsaka, Japan; ^4^ATR Computational Neuroscience LaboratoriesKyoto, Japan

**Keywords:** Bayes inference, spike train, inferior olive, gap junctions, non-stationary

## Abstract

The inverse problem for estimating model parameters from brain spike data is an ill-posed problem because of a huge mismatch in the system complexity between the model and the brain as well as its non-stationary dynamics, and needs a stochastic approach that finds the most likely solution among many possible solutions. In the present study, we developed a segmental Bayesian method to estimate the two parameters of interest, the gap-junctional (*g_c_*) and inhibitory conductance (*g_i_*) from inferior olive spike data. Feature vectors were estimated for the spike data in a segment-wise fashion to compensate for the non-stationary firing dynamics. Hierarchical Bayesian estimation was conducted to estimate the *g_c_* and *g_i_* for every spike segment using a forward model constructed in the principal component analysis (PCA) space of the feature vectors, and to merge the segmental estimates into single estimates for every neuron. The segmental Bayesian estimation gave smaller fitting errors than the conventional Bayesian inference, which finds the estimates once across the entire spike data, or the minimum error method, which directly finds the closest match in the PCA space. The segmental Bayesian inference has the potential to overcome the problem of non-stationary dynamics and resolve the ill-posedness of the inverse problem because of the mismatch between the model and the brain under the constraints based, and it is a useful tool to evaluate parameters of interest for neuroscience from experimental spike train data.

## 1. Introduction

Rapid progress in computer science now enables simulations of neuronal networks with high complexity. Advanced technology in neuroscience—such as the multiple electrode arrays, optical recording using various dyes, and optogenetic techniques—enable sampling of a massive amount of neuronal data from the brain (Nikolenko et al., 2007; Pastrana, 2010). Combining technologies across two fields of science to understand the computations in the brain still face severe difficulties mainly because of the fact that the technologies of both fields are still rather simplistic compared to a huge complexity of the brain network. It is nevertheless a big challenge in computational neuroscience to construct a brain model that simulates brain computations.

Modeling of the brain requires a number of parameters that are difficult to measure using current technology. Various approaches have been developed to resolve this “parameter estimation” problem. There are deterministic approaches that find unique solutions by optimization techniques, including the conjugate gradient, genetic algorithm, simulated annealing, and random search methods (Kirkpatrick et al., 1983; Vanier and Bower, 1999; Keren et al., 2005). These methods are only applicable in relatively well-defined environments where the complexity of the system—such as the hierarchy, granularity, and degrees-of-freedom—is comparable between the model and experiment. Otherwise, parameter estimation problems become ill-posed. Another deterministic approach uses state and parameter reconstruction based on rather simplified neural models, such as Hindmarsh–Rose and FitzHugh–Nagumo models (Fairhurst et al., 2010; Tyukin et al., 2010). Stochastic approaches were developed to overcome these difficulties, e.g., Markov random field model that estimates membrane resistance from the optical imaging data (Kitazono et al., 2012) and stochastic models that estimate the synaptic conductance from the electrophysiological recording data (Berg and Ditlevsen, 2013). Nonlinear state space modeling has also been applied to estimate hidden dynamical variables as well as unknown parameters from the optical recording data (Tsunoda et al., 2010; Meng et al., 2011). These approaches were also limited to the cases of a small mismatch between the model and experiment where the system complexity for the two cases was almost comparable. Another difficulty is that brain dynamics are non-stationary. Neuronal firing in the brain exhibits various types of irregularity in its dynamics (Ikeda et al., 1989; Kaneko, 1990; Tsuda, 1992; Tsuda et al., 2004) that are difficult to model.

The present study aims to estimate the conductance of the inferior olive (IO) neurons from spike train data using the network model simulation, which is confronted by various mismatch problems in the system complexity between the model and experimental data. The first is the granularity-hierarchy mismatch. The experimental spike data are generated by the network while the parameters to be estimated exist at the synapses. The second is the degrees of freedom mismatch. The real IO conveys far more complicated structures with huge degrees of freedom than those for the model, the number of IO neurons being at least four orders of magnitude greater than that for the model. The third mismatch is that IO firing dynamics are highly non-stationary, showing chaos, oscillations and other non-stationary properties (Schweighofer et al., 2004), while those of the model convey rather low non-stationarity. Therefore, we cannot expect that the network model can perfectly simulate the experimental data, and no one-to-one mapping would hold between the experimental data and the model parameters. None of the current approaches, either deterministic or stochastic, would be suitable for resolving this huge mismatch problem.

A previous study (Onizuka et al., 2013) resolved this huge mismatch problem by combining the deterministic approach with the statistical one in two ways. First, the model parameters to be fitted to the experimental data were estimated in a deterministic fashion as those of simulation data with the minimum distance to the experimental ones. Nevertheless, this procedure can be regarded as an extreme case of a class of statistical Bayesian estimation algorithms where a variance of a mixture-of-Gaussian model to translate spike data to the parameter values is assumed to be infinitely small. Second, the experimental spike data for every neuron were divided into short segments and the parameters were estimated for each segment. Then, these parameter values were pooled to give the probability distribution of the parameter values for the entire neuronal data, thus introducing the statistical estimates. The aim of the current study is to present a general framework based on a hierarchical Bayesian inference, adopting the same estimation problem of the two conductance values in IO network as used in our previous study. Our method estimates conductance values for spike segments using the forward models generalized to the entire spike data and merges the segmental estimates into a single estimate for every neuron. The segmental Bayes is equivalent to the method used in the recent studies that introduced the system noise in order to reduce the estimation errors due to modeling errors (Arridge et al., 2006; Huttunen and Kaipio, 2007; Kaipio and Somersalo, 2007). To allow segmental fluctuations in the parameter estimates and to merge the estimates for a single neuron imply to assume noise for parameter estimation with the constraint to minimize fluctuations within single neurons. This neuronal constraint avoids over-fitting of the forward models to experimental data that was the case in the previous study, reducing the number of Gaussians by three orders of magnitude, and the fitting errors to less than one-third of those in previous studies with highly non-stationary data.

## 2. Methods

### 2.1. Experimental data

We used the same spike data as those for a previous study (Onizuka et al., 2013). They include the spike data collected from two picrotoxin (PIX; Lang et al., 1996; Lang, 2002) and one carbenoxolone (CBX; Blenkinsop and Lang, 2006) studies. These studies sampled the IO spike as the complex spikes of Purkinje cells and blocked the inhibitory and gap-junctional conductance (*g_i_* and *g_c_*) of IO neuronal circuitry by application of PIX and CBX. PIX and CBX experiments contained the spike data of 500-s-long samples from 136 and 35 neurons, respectively.

### 2.2. Conductance parameters estimation

It has been shown that the inhibitory synaptic conductance (*g_i_*) and the gap-junction conductance (*g_c_*) are the two major determinants of the IO firing (Llinas et al., 1974; Llinas and Yarom, 1981; Best and Regehr, 2009). Our goal was to inversely estimate these conductance values from the IO firing data.

This inverse problem contains two practical difficulties. One is that it is an ill-posed problem because of the fact that IO firing dynamic depends on the ratio of *g_i_* and *g_c_* rather than their actual values and the other is because of the highly non-stationary and complicated firing dynamics that are difficult for current IO network models to precisely simulate. To overcome these difficulties, we tested a segmental Bayesian method.

The first difficulty is resolved by introducing a neuronal commonality constraint such that *g_c_* remains unchanged between PIX and control (CON) conditions, whereas *g_i_* remains unchanged between CBX and CON conditions in one neuron. The second difficulty was resolved by dividing whole spike trains into short time segments, estimating the parameters in each segment, and then integrating them into a single value according to hierarchical Bayesian inference.

In the segmental Bayesian method, the following six steps were applied to each neuron's data (EXP) to estimate the conductance values (*g_i_*, *g_c_*) of each neuron:

1. The network of IO neurons is simulated to generate spike trains (SIM data).
2. We evaluated the IO firing dynamics in short time segments in terms of a feature vector (FV) composed of multiple quantities such as mean firing rate, auto- and cross-correlation, local variation (LV), minimal distances (MDs), and spike distance (SD).
3. We transformed the FVs into low dimensional principal components according to feature extraction based on mutual information and principal component analysis (PCA).
4. The likelihood function was estimated as a forward model using the Gaussian mixture model in PCA space based on the SIM data.
5. The likelihood of EXP data for segments was calculated.
6. Finally, single *g_i_* and *g_c_* values for the whole experimental data in one neuron was estimated by a hierarchical Bayesian inference, where a neuronal commonality constraint was imposed as a hierarchical prior and the variability of *g_i_* and *g_c_* in segments was represented as the model variance.

#### 2.2.1. IO neuronal network simulation

**Figure 1:**
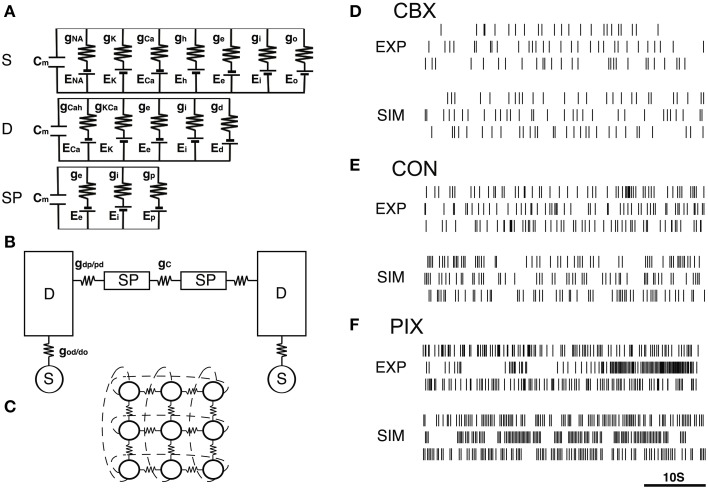
**IO network model and representatives of EXP and SIM spike trains. (A–C)** A schematic diagram of the IO network model consisting of 9 neurons, each of which consists of the soma (S), dendrite (D) and spine compartments (SP). The S, D, and SP compartments contain five, three, and one ionic channels defined by the modified Hodgkin–Huxley equations (cf. Equations A1–A17, Onizuka et al., 2013) and the excitatory and inhibitory input conductance (*g_e_* and *g_i_*). Two neighboring neurons are coupled via gap junctional conductance (*g_c_*) and axial spine conductance (*g*_dp/pd_). **(D–F)** Three representative pairs of EXP and SIM spike segment (50 s) that showed the closest match in the 3D PCA space constructed of the 25 FVs for the CBX, CON, and PIX conditions. EXP spike segments in each condition are of different neurons.

The model was composed of 3×3 neurons (Figure 1C), each of which consists of a soma, dendrite, and spine compartments (Figure 1A). The neurons were connected to each other via gap-junctions (Figure 1B). We simulated the IO firing according to the equations representing the equivalent circuitry summarized in Figures 1A–C (cf. Equations A1–A17, Onizuka et al., 2013).

The soma, dendrite and spine compartments received the excitatory and inhibitory inputs through 10, 80, and 10 synapses, respectively, driven by Gaussian noise generators. Synaptic noise was used to produce spatiotemporal dynamics in the simulation spike trains. We found that the length used for the simulation data in the previous study (500 s) was insufficient to cover the spatio-temporal dynamics of the IO spike data (cf. **Figures 3A–C**), and therefore generated 10× longer (e.g, 5000 s) simulation data.

Several studies have shown that IO neurons covey heterogeneity in their membrane conductance (Manor et al., 1997; Hoge et al., 2011; Torben-Nielsen et al., 2012). We assumed comparable variations of *g*_Cal_ and *g_c_*, in our model, sampling them from uniform distributions with the maximum deviation set at 5% of the mean. The two parameters of concern, *g_i_* and *g_c_*, were varied in the range of [0–1.5 mS/cm^2^] and [0–2.0 mS/cm^2^], respectively, with an increment of 0.05 mS/cm^2^, whereas the excitatory conductance *g_e_* was fixed at 0.03 mS/cm^2^.

#### 2.2.2. Feature vectors

We also used the same FVs as those in a previous study adding the spike distance metric (Kreuz et al., 2013) to characterize the spatiotemporal properties of the spike train (cf. Methods, Onizuka et al., 2013). To perform the segmental Bayesian inference, we divided the experimental spike data into 10 50-s segments. For each segment, 68 features were evaluated and they were averaged across three neurons to improve the signal-to-noise ratio. The first three classes of the FV represent temporal properties, while the last three represent spatial properties of the firing patterns.

1. The mean firing rate (FR) of spike segments was calculated as the number of spikes in 1 s.
2. The local variation (LV) was calculated as
  (1) $$LV=\frac{1}{R-1}\sum_{r\;=\;1}^{R-1}\frac{3{\left(T_{r+1}-T_{r}\right)}^{2}}{{\left(T_{r+1}+T_{r}\right)}^{2}}$$
  where *T_r_* (*r* = 1, 2…*R*) is the *r*-th inter-spike interval (ISI) (Shinomoto et al., 2005).
3. The auto-correlogram for 20 delays (ACG 1–20) ranged from 0 to 1000 ms with a bin size of 50 ms.
  (2) $$ACG_{x,i}(\tau )=\sum_{k\;=\;1}^{K}x_{i}(t_{k})x_{i}(t_{k}-\tau )$$
  where *x_i_*(*t_k_*) represents the occurrence of spikes at the *k*-th time step in *i*-th neuron and τ is the time delay.
4. The cross-correlogram for 20 delays (CCG 1–20) corresponding to delays of 0–50 ms, 50–100 ms, …c 950–1000 ms were computed as:
  (3) $$CCG_{x,i,j}(\tau )=\sum_{k\;=\;1}^{K}x_{i}(t_{k})x_{j}(t_{k}-\tau )$$
5. The minimal distance (Hirata and Aihara, 2009) (MD1–25) was defined as a normalized distribution of
  (4) $$s_{l}^{i,j}=1-\exp\left(\frac{-2\min_{m}|t_{l}^{i}-t_{m}^{j}|}{{\bar{d}}_{j}}\right)$$
  between the *l*-th spike of neuron *i* and a spike of neuron *j*. Here, *t_l_*^*i*^ is the time of *l*-th spike of the neuron *i*, *d*_*j*_ is the mean ISI of the *j*-th neuron, and *m* ranged from 1 to the total number of spikes of neuron *j*. If the spike train is generated by a random process, the distribution will be uniformly distributed between 0 and 1.
6. The spike distance (Kreuz et al., 2013) (SD) was defined as
  (5) $$D_{S}=\frac{1}{T}\int_{t\;=\;0}^{T}S(t)\text{d}t$$
  where *S*(*t*) are instantaneous dissimilarity values derived from differences between the spike times of the two spike trains and *T* is the recording time. SD is bounded in the range [0, 1] with the value zero obtained for perfectly identical spike trains.

#### 2.2.3. Mutual information and feature extraction

As described in Section 2.2.2, a total of 68 features were computed from the firing data. To estimate the conductance parameters efficiently, only the main features that contain rich information on *g_i_* and *g_c_* were extracted from the FV according to the mutual information (MI) between the FV and the conductance values.

We let *g* = (*g_i_*, *g_c_*) ∈ *G* as a pair of the conductance parameters and *x* ∈ *X* denote one component of the FV. Then, the MI that represents the amount of information of *G* conveyed by *x* was computed as:

(6) $$\begin{array}{l} I(G;x)=\sum_{g\in G}P(x,g)\log\left(\frac{P(x,g)}{P(x)P(g)}\right)\\ \;=\sum_{g\in G}P(x|g)P(g)\log\left(\frac{P(x|g)}{P(x)}\right) \end{array}$$

Here, the conditional distribution *P*(*x*|*g*) was approximated as a histogram of *x* given a pair of (*g_i_*, *g_c_*), the distribution *P*(x) was assumed to be a histogram of *x*, and *P*(g) was assumed to be a uniform distribution. The 68 FVs were rated by the MI and top 25 FVs were selected for principal component analysis.

#### 2.2.4. Principal component feature vectors

To reduce the redundancy further, principal component analysis (PCA) was conducted as a solution to the following equation:

(7) $$(X^{T}X)W=\lambda W,$$

where (***X***^***T***^ ***X***) is the covariance matrix of the 25 features of EXP spike data ***X***. We used a Statistical Toolbox (MATLAB®) to calculate the eigenvector ***W*** and eigenvalues λ. The principal component vector ***Y*** was computed as the linear transformation of the feature vector ***X*** as follows:

(8) Y=XW

Finally, the top three principal components of ***Y*** were selected according to the highest eigenvalues (0.27, 0.21 and 0.09) for construction of the forward model.

#### 2.2.5. Forward model

To evaluate the fitting between the experimental and simulation data, we constructed a forward model as a likelihood function in PCA space using the simulation data. The likelihood functions at each grid point of *g* = (*g_i_*, *g_c_*) were approximated by Gaussian mixture models:

(9) $$P(y|g)=\sum_{k\;=\;1}^{K}\pi_{k}N(\mu_{k},\Sigma_{k}),$$

where *N*(μ, Σ) is the multivariate Normal (Gaussian) distribution with mean μ and covariance Σ. The number of components *K*, mixing coefficients π*_k_*, means μ*_k_* and covariance matrices Σ*_k_* of Gaussian mixtures were estimated from the simulated data for a given parameter set *g* using the variational Bayes algorithm (Sato, 2001; Chapter 9 of Bishop, 2006). The average number of component *K*, which was automatically determined by the algorithm, was 8.55. The performance of the fitting was confirmed by comparing the PC scores of SIM with that predicted by the forward model by the statistical energy test (Aslan and Zech, 2005).

#### 2.2.6. Segment-wise neuronal bayesian model

Given the principal component features *y* as described in Section 2.2.4 (Equation 8), we needed to estimate the conductance parameters *g_i_* and *g_c_* that generated the corresponding firing dynamics of the IO neurons. Our model can be considered as a kind of hierarchical Bayesian model (Chapter 5 of Gelman et al., 2013), which consists of three probability distributions: the likelihood function, prior distribution and hyper-prior distribution. The likelihood function was obtained as shown in Section 2.2.5. The prior and hyper-prior distributions function as the constraints for the Bayesian estimation. The model parameters were finally estimated by computing the posterior distribution and the model evidence.

- Bayesian model with the commonality constraint
  First, a commonality constraint was introduced based on the fact that PIX and CBX selectively reduce *g_i_* and *g_c_*, respectively. This implies that *g_c_* remains unchanged between the PIX and CON conditions, whereas *g_i_* remains unchanged between the CBX and CON conditions. The commonality constraint thus assumes that PIX and CON data share the same conductance value for *g_c_* in a prior distribution, while CBX and CON data share the same *g_i_*.
  We let *y*^CON^(*t*) and ***y***^***CON***^ = [*y^CON^*(1), *y^CON^*(2), …, *y^CON^*(*T*)] denote the feature vector for time segment *t* and the collection of the segment-wise feature vectors for the control conditions, respectively. Similarly *y*^pha^(*t*) and ***y***^***pha***^ were defined for the pharmacological condition, where *pha* stands for either PIX or CBX. In addition, we let [*g^CON^_i_*, *g^CON^_c_*, *g^pha^_i_*, *g^pha^_c_*] denote the conductance parameters for a neuron under the control and pharmacological conditions.
  Thus, the likelihood function of the model is
  (10) $$\begin{array}{l} P\left(y^{CON},y^{pha}|g_{i}^{CON},g_{c}^{CON},g_{i}^{pha},g_{c}^{pha}\right)\\ \;=P\left(y^{CON}|g_{i}^{CON},g_{c}^{CON}\right)P\left(y^{pha}|g_{i}^{pha},g_{c}^{pha}\right)\\ \;=\prod_{t}P\left(y^{CON}(t)|g_{i}^{CON},g_{c}^{CON}\right)P\left(y^{pha}(t)|g_{i}^{pha},g_{c}^{pha}\right), \end{array}$$
  where *P*(*y*|*g_i_*, *g_c_*) is the probability density function constructed from the forward model. As prior distributions, we assume uniform distributions for *g_i_* and *g_c_* with commonality constraints. In the case of *pha* = PIX, *g^CON^_c_* = *g^PIX^_c_*, thus
  (11) $$\begin{array}{l} P_{0}\left(g_{i}^{CON},g_{c}^{CON},g_{i}^{pha},g_{c}^{pha}\right)\\ \;=P_{0}\left(g_{i}^{CON},g_{i}^{PIX},g_{c}^{CON}\right)\delta \left(g_{c}^{CON}-g_{c}^{PIX}\right)\propto \delta \left(g_{c}^{CON}-g_{c}^{PIX}\right) \end{array}$$
  where δ (*g_c_*) is the Dirac delta function. In the case of *pha* = CBX
  (12) $$\begin{array}{l} P_{0}\left(g_{i}^{CON},g_{c}^{CON},g_{i}^{pha},g_{c}^{pha}\right)\\ =P_{0}\left(g_{c}^{CON},g_{c}^{CBX},g_{i}^{CON}\right)\delta \left(g_{i}^{CON}-g_{i}^{CBX}\right)\propto \delta \left(g_{i}^{CON}-g_{i}^{CBX}\right) \end{array}$$
  Equations (10), (11) or (10), (12) constituted the neuronal Bayesian model.
- Hierarchical Bayesian model with the neuronal and commonality constraints
  In addition to the commonality constraint, a neuronal constraint was introduced. This constraint dealt with the estimation errors caused by the non-stationarity of the IO dynamics as well as by the incapability of the model to faithfully reproduce the complicated firing patterns of the experimental data. To minimize such errors, we divided the spike data of each neuron into segments, applied the above Bayesian model to estimate *g_i_* and *g_c_* for every segment, and then merged the segmental estimates into a single estimate for each neuron. In this framework, the estimation errors from non-stationarity can be treated as the variance of the estimates.
  This idea can be implemented by expanding the Bayesian model to a hierarchical one that employs an additional hierarchical prior distribution for merging the segmental estimates. In this model, each segment of data is generated from a segment-wise conductance parameters [*g^CON^_i_(t)*, *g^CON^_c_(t)*, *g^pha^_i_(t)*, *g^pha^_c_(t)*] which vary around neuronal conductance parameters [*g^CON^_i_*, *g^CON^_c_*, *g^pha^_i_*, *g^pha^_c_*]. The variations in the conductance parameters are considered to reflect discrepancies between the simulation dynamics and the complex dynamics of real neurons.
  Thus, the likelihood function became
  (13) $$\begin{array}{l} P\left(y^{CON},y^{pha}|g_{i}^{CON}(1:T),g_{c}^{CON}(1:T),g_{i}^{pha}(1:T),g_{c}^{pha}(1:T)\right)\\ \;=P\left(y^{CON}|g_{i}^{CON}(1:T),g_{c}^{CON}(1:T)\right)P\left(y^{pha}|g_{i}^{pha}(1:T),g_{c}^{pha}(1:T)\right)\\ \;=\prod_{t}P\left(y^{CON}(t)|g_{i}^{CON}(t),g_{c}^{CON}(t)\right)P\left(y^{pha}(t)|g_{i}^{pha}(t),g_{c}^{pha}(t)\right), \end{array}$$
  where *g^CON^_i_*(1 : *T*) = [*g^CON^_i_*(1), *g^CON^_i_(2)*, …, *g^CON^_i_* (*T*)] are collections of the segment-wise conductance, and similarly, for *g^CON^_c_*(1 : *T*), *g^pha^_i_*(1 : *T*) and *g^pha^_c_*(1 : *T*).
  We assume segment-wise conductance parameters vary around neuronal conductance parameters following Gaussian distribution with unknown variance parameters. Thus, the prior distribution became
  (14) $$\begin{array}{l} P\left(g_{i}^{CON}(t),g_{c}^{CON}(t),g_{i}^{pha}(t),g_{c}^{pha}(t)|g_{i}^{CON},g_{c}^{CON},g_{i}^{pha},g_{c}^{pha}\right)\\ \;=\prod_{t}P\left(g_{i}^{CON}(t),g_{c}^{CON}(t),g_{i}^{pha}(t),g_{c}^{pha}(t)|g_{i}^{CON},g_{c}^{CON},g_{i}^{pha},g_{c}^{pha}\right) \end{array}$$
  (15) $$\begin{array}{l} P\left(g_{i}^{CON}(t),g_{c}^{CON}(t),g_{i}^{pha}(t),g_{c}^{pha}(t)|g_{i}^{CON},g_{c}^{CON},g_{i}^{pha},g_{c}^{pha}\right)\\ \;=\{\begin{array}{ll} N\left(\left[\begin{array}{cc} g_{i}^{CON} & g_{c}^{CON} \end{array}\right],\left[\begin{array}{cc} \sigma_{1} & 0\\ 0 & \sigma_{2} \end{array}\right]\right)N\left(\left[\begin{array}{cc} g_{i}^{PIX} & g_{c}^{PIX} \end{array}\right],\left[\begin{array}{cc} \sigma_{3} & 0\\ 0 & \sigma_{2} \end{array}\right]\right) & \\ \text{ if }pha\;=\text{PIX} & \\ N\left(\left[\begin{array}{cc} g_{i}^{CON} & g_{c}^{CON} \end{array}\right],\left[\begin{array}{cc} \sigma_{1} & 0\\ 0 & \sigma_{2} \end{array}\right]\right)N\left(\left[\begin{array}{cc} g_{i}^{CBX} & g_{c}^{CBX} \end{array}\right],\left[\begin{array}{cc} \sigma_{1} & 0\\ 0 & \sigma_{3} \end{array}\right]\right) & \\ \text{ if }pha\;=\text{CBX} & \end{array} \end{array}$$
  Under the assumption of commonality constraints, *g_c_* distributions of PIX and CON shared the same variance σ_2_, and *g_i_* distributions of CBX and CON shared the unique variance σ_1_ (Equation 15). Equations (13–15) constitute the segment-wise neuronal Bayesian model, which has hierarchical prior distributions.
  Finally the commonality priors as given by (11) or (12) are assumed in the hyper-prior distribution. Note that this model is equivalent to the neuronal Bayesian model (Equations (10), (11) or (10), (12)) above when all σ_1_, σ_2_, and σ_3_ are fixed to zeros.
- Inference of conductance parameters and variance parameters
  Given the variance parameters, the conductance values can be inferred by computing the posterior distribution of the hierarchical Bayesian model above. The posterior distribution for the four conductance parameters for a neuron is given as:
  (16) $$\begin{array}{l} P\left(g_{i}^{CON},g_{c}^{CON},g_{i}^{pha},g_{c}^{pha}|y^{CON},y^{pha}\right)\\ \;=\frac{P\left(y^{CON},y^{pha}|g_{i}^{CON},g_{c}^{CON},g_{i}^{pha},g_{c}^{pha}\right)P0\left(g_{i}^{CON},g_{c}^{CON},g_{i}^{pha},g_{c}^{pha}\right)}{P\left(y^{CON},y^{pha}\right)} \end{array}$$
  Here, the numerator, the likelihood distribution integrated across all segments, is given by:
  (17) $$\begin{array}{l} P\left(y^{CON},y^{pha}|g_{i}^{CON},g_{c}^{CON},g_{i}^{pha},g_{c}^{pha}\right)\\ \;=\int \int \int \int P\left(y^{CON},y^{pha}|g_{i}^{CON}(1:T),g_{c}^{CON}(1:T),g_{i}^{pha}(1:T),g_{c}^{pha}(1:T)\right)\\ \;P\left(g_{i}^{CON}(1:T),g_{c}^{CON}(1:T),g_{i}^{pha}(1:T),g_{c}^{pha}(1:T)|g_{i}^{CON},g_{c}^{CON},g_{i}^{pha},g_{c}^{pha}\right)\\ \text{ d}g_{i}^{CON}(1:T),\text{d}g_{c}^{CON}(1:T),\text{d}g_{i}^{pha}(1:T),\text{d}g_{c}^{pha}(1:T) \end{array}$$
  and the denominator, called the model evidence, is given by:
  (18) $$\begin{array}{l} P\left(y^{CON},y^{pha}\right)=\int \int \int \int P\left(y^{CON},y^{pha}|g_{i}^{CON},g_{c}^{CON},g_{i}^{pha},g_{c}^{pha}\right)\\ \;P_{0}\left(g_{i}^{CON},g_{c}^{CON},g_{i}^{pha},g_{c}^{pha}\right)\text{d}g_{i}^{CON},\text{d}g_{c}^{CON},\text{d}g_{i}^{pha},\text{d}g_{c}^{pha} \end{array}$$
  In general, these integrals are very difficult to evaluate. However, since in our problem, the domain of (*g_i_*, *g_c_*) is discretized with bins of 0.05 and the probability mass is assumed on the grid points, the integrals appearing in Equations (17), (18) were replaced by summation and could be numerically evaluated without difficulty. Here, the conductance parameters were estimated as the maximizer of the posterior distribution.
- Inference of the variance parameters
  The variance parameters were adjusted based on the model evidence value *P*(***y^CON^***,***y^pha^***) for each neuron. We discretized the space of the possible variance parameters with a bin size of 0.025, computed the evidence (Equation 18) for all the combinations of σ_1_, σ_2_ and σ_3_, and then selected those that maximized the model evidence value.

#### 2.2.7. Differences from our previous approach

In this subsection, we briefly explain the main differences between the current approach and our previous method (Onizuka et al., 2013). In our previous method, the parameter estimation for an experimental spike train in a short time segment was given a best fit by *g* = (*g_i_*, *g_c_*) with which the error between the experimental and simulation data in PCA space was minimal over all of the generated simulation data. From the Bayesian viewpoint, this can be interpreted as a maximum likelihood estimation with the following Gaussian mixture likelihood function *P*(*y*|*g*):

(19) $$P(y|g)=\sum_{n=1}^{N_{s}}\frac{1}{N_{s}}N(y_{n}(g),\sigma^{2}I)\approx C\exp\left(-\frac{1}{2\sigma^{2}}\min_{n}{\left(y-y_{n}(g)\right)}^{2}\right),$$

where *y_n_*(*g*) is the *n*-th simulation sample at (*g_i_*, *g_c_*), *N_s_* is the total number of simulation samples (*n* = 12,600) at (*g_i_*, *g_c_*) and *C* is the normalization constant. Here, the variance σ^2^ is assumed to be infinitesimally small. This forward model is highly dependent on the generated simulation data and tends to over-fit the experimental data. The average component number *K* for the present case was roughly three orders of magnitude smaller than that for Onizuka's case (8.55:12,600), indicating the existence of this over-fitting in the latter case. Thus, our new method prevents over-fitting by explicitly estimating the smooth likelihood function using a small number of Gaussian mixtures.

In our previous study, the commonality constraint was imposed at the condition level rather than the neuronal level. Specifically, it was assumed that PIX and CON data share the same *g_c_*, whereas CBX and CON data share the same *g_i_* across the whole data set including different animals. In the current study, we assumed a more biologically reasonable commonality constraint at the neuronal level: (*g_i_*, *g_c_*) in that different time segments were common to each neuron and the PIX and CON data share the same *g_c_*, whereas CBX and CON data share the same *g_i_* in each neuron.

### 2.3. Data analysis

#### 2.3.1. Sensitivity analysis of feature vectors

Sensitivity analysis was conducted to evaluate how the FVs sense *g_i_* and *g_c_* as the partial differential of FV with respect to the *g_i_* and *g_c_*, e.g., $\frac{\partial FV}{\partial g_{i}}$ and $\frac{\partial FV}{\partial g_{c}}$. We constructed a 3D map for each FV as a function of *g_i_* and *g_c_*, by normalizing FV by the peak value. The sensitivity was determined as the mean of the partial differentials across the entire range of *g_i_* or *g_c_*.

#### 2.3.2. Non-stationary analysis

We evaluated the non-stationarity of IO firing dynamics by three measures, including LV [cf. Equation (1)], Kolmogorov–Smirnov (KS) distance of the inter-spike intervals (ISIs) to the Poisson model, and the standard deviation of the firing frequency.

## 3. Results

### 3.1. Network simulation and spike train analysis

Figures 1D–F show representative pairs of the EXP for the three experimental conditions (CBX, CON, and PIX) and the corresponding SIM spikes that were generated by the *g_i_* and *g_c_* values estimated for those spikes. A total of roughly 16,000,000 spike data trains were generated for 31 × 41 combinations of *g_i_* and *g_c_* values each for 5000 s to cover the spatiotemporal dynamics of the IO experimental (EXP) spike data (cf. **Figures 3A–C**).

### 3.2. Feature estimation

**Figure 2:**
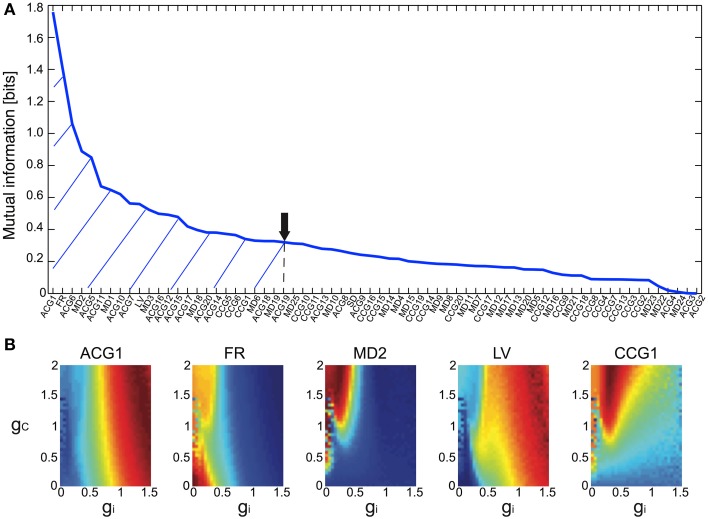
**Mutual information of the 68 FVs and maps of five major features for** ***g_i_*** **and** ***g_c_***. **(A)** Mutual information concerning *g_i_* and *g_c_* values plotted in bits for the 68 FVs. Hatch and downward arrow indicate the 25 FVs selected for PCA. **(B)** 3D maps of the five representative FVs (ACG1, FR, MD2, LV, CCG1) plotting the mean of FVs for *g_i_* and *g_c_* by pseudo-color representation. The color-map is normalized to the peak values.

The Bayesian inference requires a forward model that is compact and still informative of *g_i_* and *g_c_*. We tentatively selected 68 FVs—including FR, LV, SD, ACGs, CCGs, and MDs—and conducted the mutual information (MI) analysis concerning *g_i_* and *g_c_* to select the FVs (Figure 2A and Table 1). ACG1 conveyed the highest information (1.76 bits) and FR the next highest (1.41), whereas MD2, LV, and CCG1 conveyed rather small information (0.89, 0.56, and 0.34 bits, respectively). We selected the delay time for ACG and CCG around their oscillatory peaks, which may represent the time courses of auto- and cross-interaction within and across the cells (ACG1, 50 ms; CCG1, 50 ms; etc.). Sensitivity analysis indicated that some FVs (ACG1, FR in Figure 2B) were only sensitive to *g_i_*, whereas others (MD2 and LV) were sensitive to both *g_i_* and *g_c_*. This is probably due to the fact that *g_i_* controls firing in the individual cells, while *g_c_* controls interaction across the cells. The results indicate that FVs convey variable information concerning *g_i_* and *g_c_*, and we need to select only those conveying significant information concerning *g_i_* and *g_c_* for construction of the forward model, eliminating those conveying poor information.

**Table 1:** **Sensitivity of top ranked 25 FVs to changes of** ***g_i_*** **and** ***g_c_***.

**FV**	**Rank**	**MI**	**Sensitivity**		**FV**	**Rank**	**MI**	**Sensitivity**	
			***g_i_***	***g_c_***				***g_i_***	***g_c_***
ACG1	1	1.76	+++	n	ACG15	14	0.48	+	+
FR	2	1.41	+++	n	ACG17	15	0.42	+	+
ACG6	3	1.06	++	+	MD18	16	0.40	+	+
MD2	4	0.89	++	+	ACG20	17	0.38	+	+
ACG5	5	0.85	++	n	ACG14	18	0.38	+	+
ACG11	6	0.67	+	+	CCG5	19	0.37	+	+
MD1	7	0.65	+	+	CCG6	20	0.36	+	+
ACG10	8	0.62	++	+	CCG1	21	0.34	+	+
ACG7	9	0.56	+	+	MD6	22	0.33	++	+
LV	10	0.56	+++	+	ACG18	23	0.33	+	+
MD3	11	0.52	+	+	MD19	24	0.33	+	+
ACG16	12	0.50	+	+	ACG19	25	0.32	+	+
ACG12	13	0.49	+	+					

*List of top 25 FVs ranked by mutual information (MI) and its sensitivity to the changes of *g_i_* and *g_c_* which indicated by n/+/++/+++ for non/low/medium/high sensitivity, respectively*.

We selected top three PCA axes to construct the forward models for the following two reasons. First, eigenvalues were high for the first two axes (0.27 and 0.21, respectively) and sharply decreased for the third one (0.09), with the sum of eigenvalues for the top three axes amounting up to 0.57. Second, MI was accordingly high for the first two axes (1.6 and 1.1 bits, respectively), significantly reduced for the next axis (0.63) and remained rather low for the remaining axes. These findings indicate that the top three PCA axes conveyed reliable information on the *g_i_* and *g_c_*.

We also studied the effects of number of FVs for three-dimensional PCA space as the evidence for Bayesian estimation (8.06E-5 for 15 FVs, 1.05E-4 for 25 FVs, and 4.88E-5 for 35 FVs), and selected the 25 FVs that exhibited the highest evidence value. They included ACG1, FR, etc., rejecting ACG2, ACG3, SD, etc. (MI and rating, 0.0001 and 68^th^, 0.0017 and 67^th^, and 0.25 and 32^nd^, respectively). ACG1 conveyed rather high MI because it hit the first peak of ACG, but ACG2, ACG3 conveyed lower MI since they were off-focused from that peak. Those FVs were found to convey more than 70% (hatched area in Figure 2A) of the *g_i_* and *g_c_* information (downward arrow in Figure 2A).

### 3.3. Goodness of fit of the forward model

**Figure 3:**
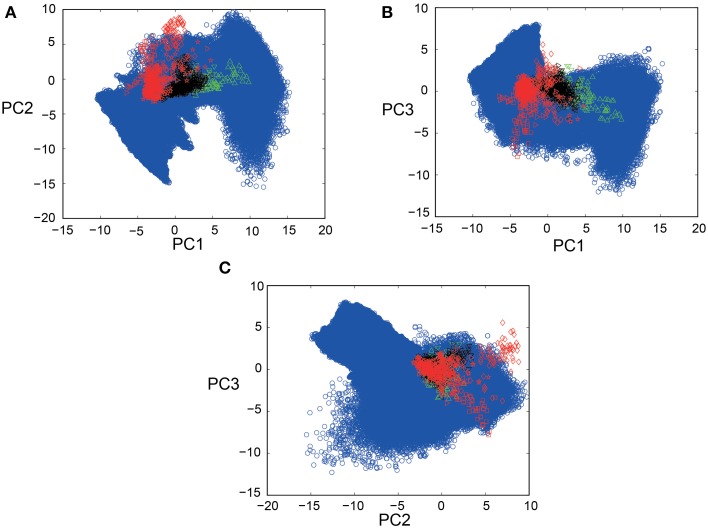
**Scatter plots of EXP and SIM spike data in 3D-PCA space. (A–C)** 2D projection views (PC1-PC2; PC1-PC3; PC2-PC3) of the scatter plots for 440, 550, and 110 spike segments for five, seven, and two animals for the PIX (red), CON (black), CBX (green) conditions, and over fifteen-million spike segments for SIM spike data (blue symbols) for 1271 combinations of *g_i_* ([0–1.5 mS/cm^2^]) and *g_c_* ([0–2.0 mS/cm^2^]). Note that the distribution of the SIM spike data perfectly covers that of the EXP data except for a fraction of the EXP data for one animal (red diamonds). The color conventions that represent the PIX, CON, and CBX conditions are the same in this and the following figures.

PCA was conducted for a total of 1100 spike segments (10 segments each for the 110 IO neurons), containing 440 segments for 44 neurons sampled in five PIX experiments, 110 segments for 11 IO neurons sampled in two CBX experiments and 550 segments for 55 IO neurons sampled in seven CON experiments. Bayesian inference requires for the forward model of SIM data to completely cover the distribution of EXP data in the PCA space. Figures 3A–C show that this requirement is satisfied by mapping SIM (blue symbols) and EXP spike data for PIX, CBX, and CON (red, green, and black symbols) into the 3D-PCA space. We confirmed that SIM spike data completely cover the distributions of EXP spike data except for a fraction of PIX data of one animal (red diamonds).

**Figure 4:**
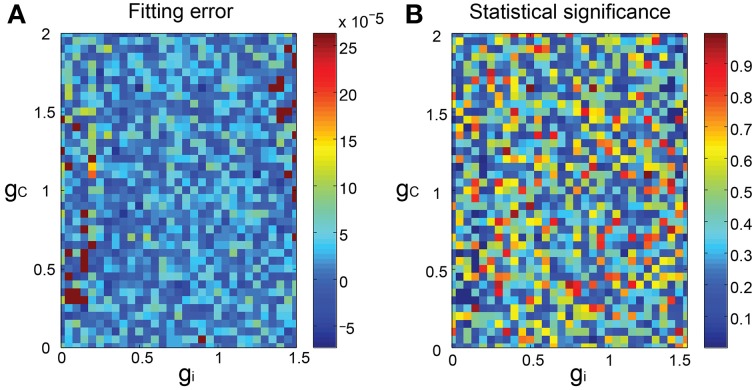
**Goodness of fit of the forward model to the SIM spike data. (A)** 3D pseudo-color representation of the fitting error of the forward model estimated as the energy test statistics between the predictions of the forward model and the SIM data plotted for *g_i_* and *g_c_*. **(B)** statistical significance of the error.

We finally constructed the forward models as mixed Gaussians fitted to the SIM spike data mapped in the PCA space and evaluated the fitting as the 3-dimensional minimum energy test of the model prediction and the SIM spike data. In general, the match was acceptable (Figure 4A), with the statistical difference being not significant (*p* > 0.1) for most combinations of *g_i_* and *g_c_*, except for few ones (about 2%) where the statistical significance was rather high (*p* < 0.03) (Figure 4B).

### 3.4. Bayesian inference with a relaxed neuronal constraint

We found that the EXP spike data were significantly non-stationary (cf. **Figures 8B–D**), which may cause errors in the Bayesian estimation of *g_i_* and *g_c_*. Those errors were minimized by the segmental Bayes whereby the entire spike data for each IO neuron was fractionated into 10 segments, Bayesian estimation of *g_i_* and *g_c_* was conducted segment by segment, under the commonality constraint that the *g_i_* estimates agree between CBX and CON, and the *g_c_* estimates between PIX and CON, respectively, and the segmental estimates were finally merged into a single estimate for every neuron (cf. Methods, Section 2.2.6) under the neuronal constraint assuming a single *g_i_* and *g_c_* for each neuron.

**Figure 5:**
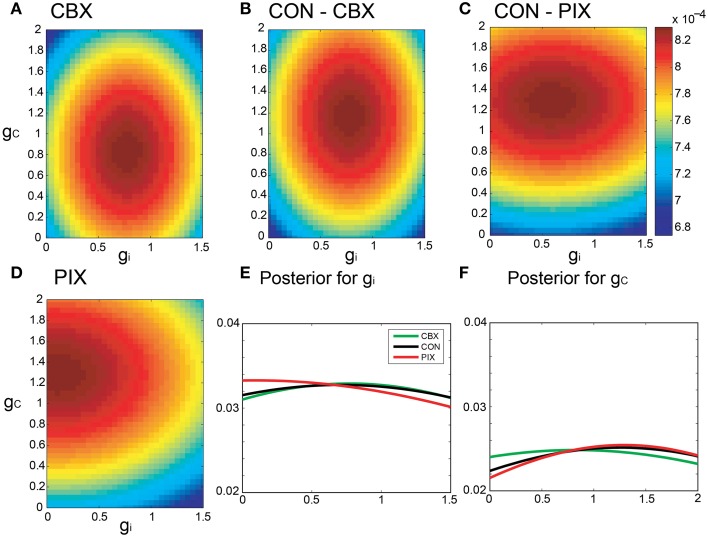
**Segmental Bayesian estimates of** ***g_i_*** **and** ***g_c_*** **with relaxed commonality constraints. (A–D)** Posterior probability of the *g_i_* and *g_c_* estimates for representative IO neurons under the relaxed commonality constraint (σ = 10) for the two experimental (CBX and PIX) and two corresponding control conditions (CON-CBX and CON-PIX). **(E,F)** The profiles of *g_i_* and *g_c_* probability of the neurons plotted in **(A–D)**.

Figures 5A–D are pseudo-color representation of the posterior probability of *g_i_* and *g_c_* estimated for a representative IO neuron by the Bayesian inference under the commonality and the relaxed neuronal constraint (σ = 10, cf. Equation 15). The estimates were diffused broadly for all of the three conditions probably because of the fluctuations of the segmental estimates. The probability of the *g_i_* and *g_c_* estimates for the IO neuron for the three experimental conditions showed broad and overlapping distributions (Figures 5E,F).

### 3.5. Bayesian inference with a neuronal constraint

**Figure 6:**
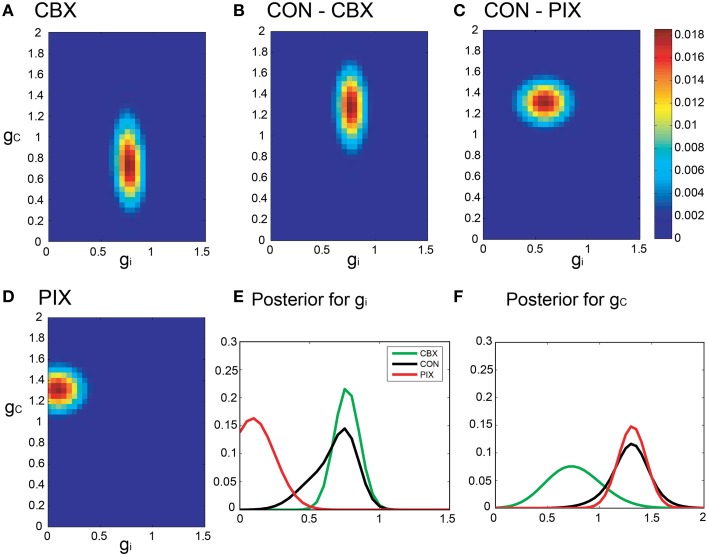
**Segmental Bayesian estimates of** ***g_i_*** **and** ***g_c_*** **with optimized commonality constraints. (A–F)** Similar posterior probability plots of *g_i_* and *g_c_* to those in Figure 5 but with the optimized commonality constraints.

**Figure 7:**
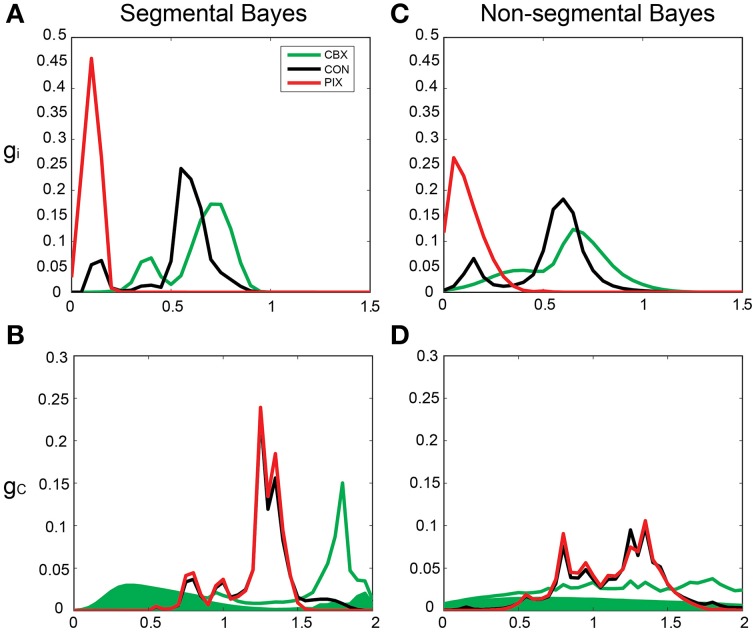
**Segmental and non-segmental Bayes estimates for the entire IO neuronal population. (A,B)** the average *g_i_* and *g_c_* estimates by the segmental Bayesian inference for the entire IO neuronal population. **(C,D)** those similar to **(A,B)** but by non-segmental Bayesian inference where the posterior probability was estimated across the entire spike train of the individual IO neurons. Filled and open areas in green in **(B,D)** represent the estimates for the two different animals in the CBX condition.

By contrast, the *g_i_* and *g_c_* of the same IO neuron as in Figure 5 estimated by Bayesian inference under the optimized neuronal constraint (σ was optimized in the range of [0.1–0.5]) were sharper, with the peak of (*g_i_*, *g_c_*) at (0.75, 0.75 mS/cm^2^) for CBX, at (0.1, 1.3 mS/cm^2^) for PIX and at (0.75, 1.25 mS/cm^2^) and (0.6, 1.3 mS/cm^2^) for CON-CBX and CON-PIX, respectively (Figures 6A–F). Figures 7A,B show the ensemble distributions of *g_i_* and *g_c_* estimated by the segmental Bayesian inference for the entire population of IO neurons in comparison with those by the non-segmental Bayes whereby *g_i_* and *g_c_* were estimated at once across the entire length of spike data (Figures 7C,D). The *g_i_* and *g_c_* estimates by the segmental Bayes essentially agreed with those by the non-segmental Bayes with the tendency for the segmental Bayesian inference to give a sharper distribution than the non-segmental Bayes. The *g_i_* value peaked at 0.6–0.7 mS/cm^2^ for CBX and CON and at 0.1–0.2 mS/cm^2^ for PIX (Figures 7A,C) and the *g_c_* for PIX and CON was 1.3 mS/cm^2^. The *g_c_* value for CBX was distributed diffusely across a wide range. We noted that the segmental Bayes gave rather conflicting estimates of *g_c_* between the two animals. In one animal there was a marked leftward shift of the peak between the CBX and CON conditions (with a reduction of *g_c_*, filled area in Figure 7B) and conversely a significant rightward shift in the other animal (open area). The non-segmental Bayesian inference also showed the same results, although the difference was less clear. The same tendency was also found in our previous study (cf. Figure 7 in Onizuka et al., 2013). Therefore this may represent reality rather than Bayesian artifacts.

**Figure 8:**
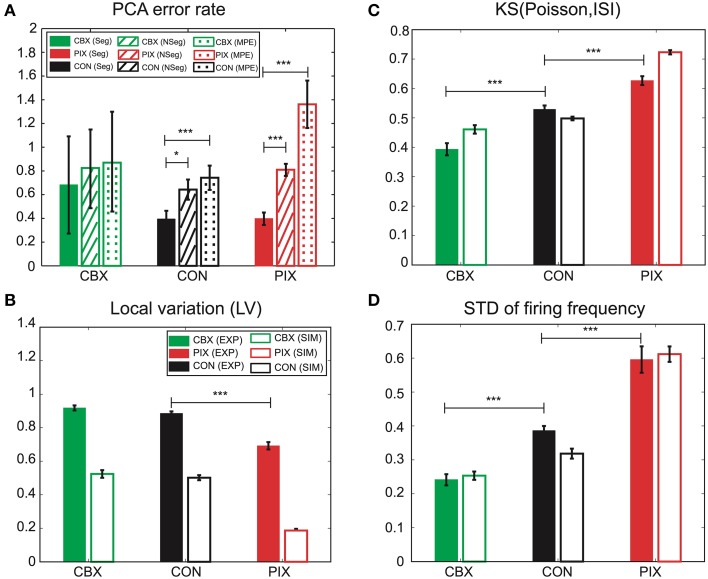
**Performance of the segmental and non-segmental Bayesian inference and the minimum PCA error method and the non-stationarity of EXP and SIM spike data. (A)** PCA error rates of the *g_i_* and *g_c_* estimates for the segmental (solid columns, Seg) and non-segmental (hatched, NSeg) Bayesian inference and the minimum PCA error method (dotted, MPE) averaged for the entire IO neurons for CBX, CON, and PIX conditions. The colors represent the three experimental conditions and the texture patterns represent the errors for the three methods of *g_i_* and *g_c_* estimates. **(B–D)** Non-stationality of the spike data estimated as the three metrics. **(B)**: LV; **(C)**: KS distance of the ISI distribution for the EXP (solid columns) and SIM (blank) spike data from Poisson distribution; **(D)** standard deviation of the instantaneous firing rate. The colors represent the three experimental conditions and the texture patterns represent EXP and SIM data. ^*^ *p* < 0.05, ^***^ *p* < 0.001.

We hypothesize that the segmental Bayes minimizes the errors of *g_i_* and *g_c_* estimation because of the failure for the forward model to reproduce the non-stationary dynamics of IO firing. This hypothesis was tested by comparing the performance of the segmental and non-segmental Bayes in terms of the PCA error rate (the difference in the PCA scores between the EXP and the corresponding SIM spikes that were generated by the *g_i_* and *g_c_* estimated for those EXP spikes). PCA errors for the segmental Bayes were smaller than the non-segmental Bayes across the three experimental conditions (*F* = 14.18, *p* = 0.0002), the difference being most significant for PIX, less for CON, and insignificant for CBX (cf. solid and hatched columns Figure 8A). Correspondingly, the non-stationarity of the EXP spikes estimated as the KS distance between the distribution of the inter-spike intervals for the EXP spikes and that of Poisson and the standard deviations of the firing rate ranked in the same order as that for the significance of the PCA error difference between the segmental and non-segmental Bayes, being high, medium and low for the PIX, CON, and CBX conditions (cf. Figures 8A,C,D), respectively. These findings are consistent with our view that the segmental Bayes minimizes errors in *g_i_* and *g_c_* estimates because of the non-stationary dynamics of IO firing. It is notable that the corresponding SIM spikes rather faithfully reproduced the non-stationarity of the EXP spikes for the two measures across the three experimental conditions, while they were significantly smaller for the LV (Figure 8B). This finding indicates that the present simulation failed to precisely reproduce the non-stationarity estimated by the LV.

Finally, we confirmed the superiority of the segmental Bayesian inference over the minimum error method used in our previous study (Onizuka et al., 2013) in terms of the PCA error rate. The magnitude of the error rate was smaller for the segmental Bayesian inference (solid columns in Figure 8A) than that for our previous study (dotted columns) across the three experimental conditions (statistical significance, *F* = 23.37, *p* < 0.0001 by ANOVA), and the statistical significance of the error rate was largest (*p* < 0.0001 by *t*-test), moderate (*p* < 0.01) and minimum (*p* > 0.7) for the PIX, CON, and CBX conditions, respectively, corresponding to the degree of the non-stationarity of the EXP spikes. It is notable that the average number of mixed Gaussians per (*g_i_*, *g_c_*) grid of the forward models was three orders of magnitude smaller in the segmental Bayes (*n* = 8.55) than that in our previous study (*n* = 12,600) and slightly larger than that for the non-segmental Bayesian inference (*n* = 2.3), indicating that there was over- and under-fitting in the previous study and the non-segmental Bayesian inference, respectively, compared with the segmental Bayesian inference used in the present study.

## 4. Discussion

The goal of the present study was to resolve the inverse problem estimating the two important parameters of the IO network (i.e., *g_i_* and *g_c_*) by fitting the firing dynamics of the model network with those of the IO network. The parameter estimation was confronted with a huge mismatch of the model network with the brain network in the system complexity such as the granularity, the hierarchy, the degrees of freedom and the non-stationary dynamics. Consequently, the inverse problem becomes severely ill-posed (Prinz et al., 2004; Achard and Schutter, 2006), and necessitates some stochastic approaches that find the most likely solution among many possible ones according to various error functions (Geit et al., 2008).

The previous study (Onizuka et al., 2013) defined the error function as the distance between the experimental and corresponding simulation spike data in the PCA space (PCA error), constructed of various feature vectors (FVs) that are derivatives of the ISIs of the experimental spike data such as the firing rate, the auto- and cross correlation, the minimum distance (MD), the spike distance (SD), and the local variance (LV), representing the spatiotemporal firing dynamics and contained strong redundancy. In that study, the FVs were determined for short spike segments (50s) to compensate for the non-stationarity of the experimental spike trains (Grun et al., 2002; Quiroga-Lombard et al., 2013), PCA was conducted to remove the redundancy, and the *g_i_* and *g_c_* were determined as the ones giving the minimum PCA errors to the experimental spike segments. The minimum error method can be regarded as the extreme case of Bayesian inference where the forward model translating the model parameters into the spike features was constructed for each spike segment. This approach is equivalent to assuming different Gaussians (i.e., parameter-spike feature translation mechanisms) for every spike segment of the same single neuron and may be regarded as over-fitting.

The present study maintained the segmental approach and corrected the over-fitting by the hierarchical Bayesian inference that estimated the *g_i_* and *g_c_* by fitting Gaussians to every spike segment and merged them into single *g_i_* and *g_c_* estimates according to the neuronal constraint that assumes the same *g_i_* and *g_c_* for a single neuron (Figures 5, 6). This view is supported by the fact that the number of Gaussians used for construction of the forward model is three orders of magnitude smaller for the present segmental Bayesian inference than that for the minimum error method.

There were additional improvements in construction of the firing feature space in two ways. First, the FVs were selected according to the mutual information of the *g_i_* and *g_c_* (Figure 2) and the number of FVs (*n* = 25) were optimized according to the evidence function. Second, the length of the simulation spike data was expanded 10 times more than that for the previous study (from 500 to 5000 s) to ensure more satisfactory reproduction of the IO firing dynamics (Figures 3, 4). The overall performance of the segmental Bayesian inference estimated as the PCA error that is the distance between the experimental and corresponding simulation spike segments in the PCA space was generally higher across the three experimental conditions than those for the minimum PCA error method and the non-segmental Bayesian inference that estimated the *g_i_* and *g_c_* across the entire spike length (Figure 8A). The statistical significance of the difference was high, modest and minimal in the PIX, CON, and CBX conditions, respectively, in correspondence with the non-stationarity of the IO firing evaluated as the three metrics, including the KS distance of the ISIs from Poisson distributions, the LV, and the standard deviation of the firing rate (Figures 8B–D). The segmental Bayes could be regarded as a way to minimize estimation errors of *g_i_* and *g_c_* due to the errors of the current forward model to precisely reproduce the non-stationality of IO firing. Allowance of fluctuations for segmental *g_i_* and *g_c_* estimates is equivalent to recent methods (Arridge et al., 2006; Huttunen and Kaipio, 2007; Kaipio and Somersalo, 2007) to reduce parameter estimation errors due to modeling errors by assuming system noise.

These findings indicate that segmental Bayesian inference performs better than the other two methods in cases of highly non-stationary firing dynamics. The estimates of *g_i_* and *g_c_* by the segmental Bayesian inference are in partial agreement with those of our previous study. The point of agreement was the *g_c_* for the CON and PIX conditions (1.21±0.2 and 1.16±0.43 mS/cm^2^ for the present and previous studies) and those of disagreement were *g_c_* for the CBX condition (1.24±0.6 and 0.75±0.51 mS/cm^2^) and *g_i_* for the CON (0.54±0.18, 1.10±0.36 mS/cm^2^), PIX (0.1±0.04, 0.51±0.41 mS/cm^2^), and CBX conditions (0.65±0.15, 1.11±0.34 mS/cm^2^). The present estimates may be more accurate than the previous ones for the three reasons. First, we expanded the *g_c_* range for simulation (from [0–1.6 mS/cm^2^] in the previous study to [0–2.0 mS/cm^2^] in the present study), second, we expanded the data length for simulation (from 500 to 5000 s) for better simulation of experimental spike dynamics, and third, the present estimates gave smaller PCA errors (0.39±0.05, 0.68±0.41, 0.39±0.07 under PIX, CBX and CON in present study and 1.36±0.20, 0.86±0.42, 0.74±0.10 in our previous study).

The *g_c_* estimates for the CBX condition diverged between two animals in the present study (Figure 7B), and the same tendency was also found in our previous study, although this tendency was less clear. The reason for this discrepancy between the two animals is unclear. CBX is a nonspecific blocker of the gap-junctional conductance and may act on other ionic conductances than the gap-junction, affecting the *g_c_* estimates. The IO units were sampled across many micro-zones of the cerebellum on which the gap-junctional conductance was dependent, being high and low in the same and different micro-zones, respectively. This heterogeneity in the *g_c_* population may also be the cause of the discrepancy.

### Conflict of interest statement

The authors declare that the research was conducted in the absence of any commercial or financial relationships that could be construed as a potential conflict of interest.
